# [Retracted] MicroRNA‑425 is downregulated in nasopharyngeal carcinoma and regulates tumor cell viability and invasion by targeting hepatoma‑derived growth factor

**DOI:** 10.3892/ol.2024.14245

**Published:** 2024-01-22

**Authors:** Wenyan Zhu, Yongchi Ma, Xuqin Zhuang, Xin Jin

Oncol Lett 15: 6345–6351, 2018; DOI: 10.3892/ol.2018.8128

Following the publication of the above article, a concerned reader drew to the Editor's attention that the Transwell cell migration and invasion assay data featured in Figs. 3B and 6C were strikingly similar to data appearing in different form in other articles written by different authors at different research institutes that had either already been published elsewhere prior to the submission of this paper to *Oncology Letters*, or were under consideration for publication at around the same time (some of which have been retracted).

In view of the fact that certain of these data had already apparently been published prior to the submission of this article for publication, the Editor of *Oncology Letters* has decided that this paper should be retracted from the Journal. The authors were asked for an explanation to account for these concerns, but the Editorial Office did not receive a reply. The Editor apologizes to the readership for any inconvenience caused.

